# Body composition, muscle strength and hormonal status in patients with ataxia telangiectasia: a cohort study

**DOI:** 10.1186/s13023-015-0373-z

**Published:** 2015-12-09

**Authors:** H. Pommerening, S. van Dullemen, M. Kieslich, R. Schubert, S. Zielen, S. Voss

**Affiliations:** Children’s Hospital, Allergology, Pneumology and Cystic Fibrosis, Goethe-University Theodor-Stern Kai, Frankfurt/Main, Germany

**Keywords:** Ataxia telangiectasia, Body composition, Muscle strength, Hormonal status

## Abstract

**Background:**

Ataxia-telangiectasia (A-T) is a devastating human autosomal recessive disorder that causes progressive cerebellar ataxia, immunodeficiency, premature aging, chromosomal instability and increased cancer risk. Affected patients show growth failure, poor weight gain, low body mass index (BMI), myopenia and increased fatigue during adolescence.

The prevalence of alterations in body composition, muscle strength and hormonal status has not been well described in classical A-T patients. Additionally, no current guidelines are available for the assessment and management of these changes.

**Methods:**

We analyzed body composition, manual muscle strength and hormonal status in 25 A-T patients and 26 age-matched, healthy controls. Bioelectrical impedance analysis (BIA) was performed to evaluate the body composition, fat-free mass (FFM), body cell mass (BCM), extracellular matrix (ECM), phase angle (PhA), fat mass (FM) and ECM to BCM ratio. Manual muscle strength was measured using a hydraulic hand dynamometer.

**Results:**

The BMI, FFM and PhA were significantly lower in A-T patients than in controls (BMI 16.56 ± 3.52 kg/m^2^ vs. 19.86 ± 3.54 kg/m^2^; Z-Score: -1.24 ± 1.29 vs. 0.05 ± 0.92, *p* <0.001; FFM 25.4 ± 10.03 kg vs. 41.77 ± 18.25 kg, *p* < 0.001; PhA: 4.6 ± 0.58° vs. 6.15 ± 0.88°, *p* < 0.001). Manual muscle strength was significantly impaired in A-T patients compared with controls (10.65 ± 10.97 kg vs. 26.8 ± 30.39 kg, *p* < 0.0001). In addition, cortisol and dehydroepiandrosterone sulfate (DHEAS) levels were significantly lower in A-T patients than in controls.

**Conclusion:**

Altered body composition, characterized by depleted BMI, PhA and BCM; by the need to sit in a wheelchair; by altered hormone levels; and by poor muscle strength, is a major factor underlying disease progression and increased fatigue in A-T patients.

**Trial registration:**

ClinicalTrials.gov NCT02345200

## Background

Ataxia telangiectasia (A-T) is an autosomal recessive genomic instability syndrome characterized by cerebellar ataxia, immunodeficiency and cancer predisposition [1–3]. Additional clinical features of A-T include oculocutaneous telangiectasias, frequent bronchopulmonary infections, growth retardation, fatigue in adolescence and premature aging [4–7]. As in other immunodeficiency and genomic instability syndromes, a high percentage of A-T patients suffer from dystrophy, stunting and poor weight gain [8–11]; however, the pathophysiology underlying A-T-mediated alterations in physical development is complex. Potential causes of failure to thrive include low growth hormone levels, hypogonadism, upper and lower respiratory system infections, catabolic bone metabolism and progressive neurodegeneration, such as dysphagia and aspiration [9, 12, 13]. Growth failure, muscle wasting and weight loss have been well described in numerous chronic diseases such as congenital heart failure, chronic kidney disease, chronic liver disease and cystic fibrosis [14, 15]. Poor growth is a common feature of A-T and may be associated with a general decline in overall health, poor caloric intake and endocrine abnormalities [8, 10, 13]. According to several studies, cachexia and impaired growth directly correlate with increased morbidity and mortality [9, 16–18]. Although they are common clinical problems in A-T patients, cachexia and myopenia have rarely been investigated.

We recently showed that the levels of circulating insulin-like growth factor-1 (IGF-1) and its main binding protein, IGF-binding protein 3 (IGF-BP3), are low in the majority of A-T patients [8, 9]. In addition to regulating somatic growth and metabolism, the growth hormone (GH)/IGF-1 axis has been implicated in regulating brain growth. Indeed, a recent study demonstrated that neurological progression was accompanied by GH/IGF-1 axis deficiency, markedly reduced body weight, high ataxia scores and advanced age [11]. Moreover, as A-T patients age, they often develop a catabolic condition that is associated with impaired glucose metabolism [3, 19]. It is tempting to speculate that decreased levels of growth hormones stemming from major endocrine dysregulation are responsible for frailty, disability, and mortality in A-T patients. However, considerable clinical variation exists among patients with A-T. The clinical phenotype of A-T is aligned with the presence of some degree of residual ATM kinase activity [20–22]; however, in our current cohort of classical A-T patients, no residual kinase activity was detected, as recently described [8].

The findings described above motivated us to conduct a detailed clinical investigation of body composition, manual muscle strength and hormonal status in 25 A-T patients and a group of healthy controls.

## Methods

Between May 2013 and April 2014, we enrolled 26 patients with A-T and 26 gender- and age-matched healthy controls for evaluation in our cross-sectional interventional study, which included one study visit. The A-T patients were clinically diagnosed according to recent World Health Organization (WHO) recommendations [23]. One A-T patient was excluded from all analysis due to lymphoma.

Body composition and muscle strength were evaluated. Hormonal status was analyzed in serum samples collected from healthy subjects ≥12 years of age and from the A-T patients.

### Eligibility

Written consent from patients or caregivers was required for each subject. The study was conducted following the ethical principles of the Declaration of Helsinki, regulatory requirements and the code of Good Clinical Practice. The study was approved by the responsible ethics committees (application number 37/13) in Frankfurt and registered at clinicaltrials.gov NCT02345200.

Comorbidities that influence body composition, such as malignoma and dialysis-dependent renal failure, were defined as exclusion criteria. Healthy controls were recruited by public posting. The subjects were matched for sex and age. Controls with any type of chronic disease were rejected. Subjects with body composition alterations out of the normal range (i.e., overweight, obesity or cachexia) were not included. Due to ethical concerns, we were not allowed to collect blood from healthy controls less than 12 years of age.

### Growth analysis and neurological examination

Weight and height were recorded, and body mass index (BMI) was calculated. Z-Scores were determined using http://aga.adipositas-gesellschaft.de/mybmi4kids/index.php. The age percentile was defined according to Cole´s least median of squares (LMS) values. Then we performed a detailed clinical neurological examination with quantification of the individual progress of ataxia by the ataxia score as recently described [11].

### Bioelectrical impedance analysis (BIA)

Bioelectrical impedance analysis (BIA) was performed using Data Input’s Nutriguard-M multi-frequency Bioelectrical Impedance Analyzer and BIANOSTIC-AT® double-size electrodes (Data Input, Pöcking, Germany) according to the manufacturer’s instructions. The current was set to 50 kHz. The subjects were required to be sober and were asked to micturate prior to measurement.

To ensure the even distribution of body water, each subject had to lie on the examination couch for 15 min. BIA calculates body compartments based on the differing conductivities of tissues with distinct biological features; the measure is proportionate to the cellular water and electrolyte contents. Impedance was measured on the dominant side from the wrist to the ipsilateral ankle using four electrodes.

The analysis was conducted using Nutri Plus software (Data Input, Pöcking, Germany). We determined the following variables: the phase angle (PhA), fat-free mass (FFM), fat mass (FM), body cell mass (BCM), extracellular matrix (ECM), and ECM/BCM ratio. Additionally, the percentage of BCM in FFM was analyzed but only for adults.

FFM is defined as the body weight minus FM; BCM and ECM together compose the FFM. BCM mainly comprises visceral proteins and intracellular water [24], whereas ECM includes bone mass and extracellular water. Software was used to calculate the ECM/BCM ratio for individuals over 15 years of age.

### Analysis of manual muscle strength

Manual muscle strength was assessed using a hydraulic hand dynamometer from BASELINE® Evaluation Instruments (Fabrication Enterprises, Inc., Elmsford, NY, USA), following the manufacturer’s guidelines. Hand position was stabilized in the A-T patients, as needed. Each subject was then asked to press the handle with maximum power before a rest period of 30 s. Each measurement was conducted in triplicate. After every measurement, the position of the indicator needle on the meter was read and then reset to zero. The mean of these three measurements was calculated.

### Hormonal status

As some hormone levels fluctuate in a circadian rhythm, blood was collected at eight in the morning. The subjects were always sober during blood collection.

A chemiluminescence immunoassay (IMMULITE 1000 Immunoassay System, Siemens, Bad Nauheim, Germany) was used to measure serum cortisol, dehydroepiandrosterone sulfate (DHEAS), GH, IGF-1, IGF-BP-3, thyroid-stimulating hormone (TSH) and vitamin D levels.

### Statistical analysis

GraphPad Prism 5.01 (GraphPad Software, Inc.) and Microsoft Excel were used for the statistical analysis. BMI, height, weight and manual muscle strength are presented as arithmetic means with standard deviations (SDs). For comparisons between the two study groups, Student’s paired t-test was applied. Correlations were analyzed by Spearman’s or Pearson’s correlation coefficient. *P*-values ≤ 0.05 were considered significant.

BMI was defined as the primary variable. The secondary variables included body compartment structures based on BIA detection, manual muscle strength and hormonal status.

## Results

Table 1 shows the characteristics of the evaluated patients. We compared 25 A-T patients and 26 gender- and age-matched healthy controls. A total of 13 female and 13 male subjects were included in the control group and 13 females and 12 males in the patient group. Of the 25 included patients, 11 (44 %) had BMIs below the 3^rd^ percentile. Two of the 25 patients (8 %) had gastrostomy tubes; both suffered from swallowing problems and had BMIs below the 3^rd^ percentile. The mean age in the A-T group was 13.04 ± 6.79 years; the mean age in the healthy control group was 14.96 ± 6.45 years. Height, weight and BMI were significantly lower in the A-T patients than in the healthy controls (Table 1).

**Table 1:** Patient characteristics

Parameters	A-T (*n* = 25)	Controls (*n* = 26)	*p*-value
Age [years]	13.04 ± 6.79	14.96 ± 6.45	
Male	12	13	
Female	13	13	
Pre-Pubertal	11 (44 %)	6 (23 %)	
Adults	7 (28 %)	11 (42.3 %)	
Height [m]	1.38 ± 0.23	1.59 ± 0.27	<0.01
Z-Score	-1.51 ± 1,53	0.35 ± 1.01	<0.01
Weight [kg]	33.38 ± 16.8	53.75 ± 23.39	<0.01
Z-Score	-1.93 ± 1.89	0.25 ± 1	<0.01
BMI [kg/m^2^]	16.56 ± 3.52	19.86 ± 3.54	<0.001
Z-Score	-1.24 ± 1.29	0.05 ± 0.92	<0.001
AFP [ng/ml]	411.8 ± 305.3		
CRP [mg/l]	5.9 ± 1.33^a^		

The data are shown as the means ± SD

^a^normal CRP levels <5 mg/l

Table 2 shows clinical and neurological characteristics of the A-T cohort. Patients were grouped in patients whose gait is still preserved and wheelchair-bound patients. Wheelchair-bound patients were older and neurologically more affected than mobile patients. In addition, they had higher alpha-fetoprotein (AFP) - values. Whereas PhA and BMI Z-Scores seem to be affected in independently from mobility, the occurrence of dysphagia, neuropathy and higher ataxia scores go along with loss of gait. In sum, the neurological impairment was increased in this group.

**Table 2:** Clinical Characteristics of mobile and wheelchair-bound patients

Parameters	Gait preserved (*n* = 10)	Wheelchair-bound (*n* = 15)	*p*-value
Age [years]	6.2 ± 2.78	17.6 ± 4.27	<0.0001
Time in wheelchair [years]		6.75 ± 3.47	
Z-Score BMI	-0.96 ± 0.89	-1.43 ± 1.55	n.s.
PhA [°]	4.8 ± 0.53	4.51 ± 0.59	n.s.
A-T-Score	12.5 ± 6.89	23.5 ± 2.58	<0.01
Dysphagia	0	10	
Neuropathy^a^	0	8	
AFP [ng/ml]	237.5 ± 154.6	436.4 ± 253.1	<0.05

The data are shown as the means ± SD

^a^Only 18 of 25 patients were investigated for peripheral neuropathy

### BIA

**Fig. 1:**
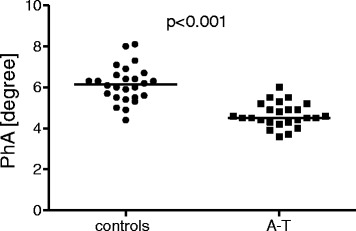
Phase angle α values in A-T patients (*n* = 25) and healthy controls (*n* = 26). The A-T patients showed significantly lower α-values than did the healthy controls; *p* < 0.001

The BIA measurements revealed significantly lower PhA values in the A-T patients than in the controls (Fig. 1; A-T 4.6 ± 0.58°, controls 6.15 ± 0.88°; *p* < 0.001). Interestingly, six of the 18 A-T patients (33.3 %) who were between two and 18 years of age had pathologically low PhA values that fell below the 3^rd^ percentile. This difference was even more pronounced in the group comprising patients over 12 years of age, in which five of the eight (62.5 %) patients had PhA values below the 3^rd^ percentile.

**Fig. 2:**
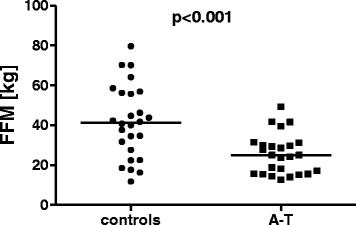
Fat free mass in A-T patients (*n* = 25) and healthy controls (*n* = 26). The A-T patients showed significantly lower FFM compared to the healthy controls; *p* < 0.001

FFM was significantly lower in the A-T patients than in the controls (Fig. 2; A-T 25.4 ± 10.03 kg, controls 41.77 ± 18.25 kg; *p* < 0.001). Four of the 18 (22.2 %) patients who were between two and 18 years of age and four of the eight (50 %) patients who were between 12 and 18 years of age had FFM values below the 3^rd^percentile. BCM, ECM and the ECM/BCM ratio were measured for all subjects over 15 years of age (12 A-T patients, 14 healthy controls).

BCM was significantly lower in the A-T patients than in the controls (A-T 14.71 ± 3.71 kg, controls 29.96 ± 8.3 kg; *p* < 0.001); the A-T patients also had significantly lower ECM levels (A-T 18.69 ± 4.38 kg, controls 24 ± 5.78 kg; *p* < 0.05). ECM was within the normal range in 11 of the 12 patients; the remaining patient had low ECM.

**Fig. 3:**
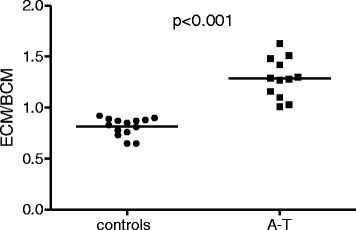
The ECM/BCM ratios of A-T patients (*n* = 12) and healthy controls (*n* = 14). The normal ECM/BCM ratio is below 0.8 for males and below 0.9 for females. The ECM/BCM ratios were significantly elevated in all patients; *p* < 0.001

The ECM/BCM ratio was significantly higher in the A-T patients than in the controls (Fig. 3; A-T 1.29 ± 0.19, controls 0.82 ± 0.09; *p* < 0.001). All 12 patients (100 %) had high ECM/BCM values.

The percentage of BCM in FFM was analyzed in only the adult subjects. Again, significantly lower levels were found in the A-T patients than in the controls (A-T 44.1 ± 4.17 %, controls 55.25 ± 2.39 %; *p* < 0.001).

### Manual muscle strength

Manual muscle strength was significantly decreased in the A-T cohort compared with the controls (A-T 10.65 ± 7.33 kg, controls 26.8 ± 19.35 kg; *p* < 0.001).

### Hormonal status

**Fig. 4:**
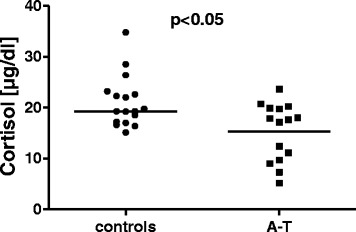
Cortisol levels in patients with A-T (*n* = 15) and healthy controls (*n* = 17). Hormone levels were measured in the ≥12 years of age group. The A-T patients showed significantly lower cortisol levels than did the healthy controls; *p* < 0.05

**Fig. 5:**
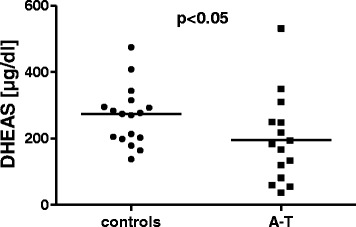
DHEAS levels in patients with A-T (*n* = 15) and healthy subjects (*n* = 17). Hormone levels were measured in the ≥12 years of age group. DHEAS levels were significantly lower in the A-T patients than in the healthy controls; *p* < 0.05

The hormone level measurements are shown in Table 3. Significantly lower cortisol, DHEAS and IGF-1 levels were found in the A-T patients compared with the controls. Unfortunately, we did not have matched controls for the younger patients. Therefore, we carefully matched the older A-T patients with controls. Significance could be confirmed for only cortisol and DHEAS levels, as shown in Figs. 4 and 5.

**Table 3:** Hormonal status

Parameters	A-T (*n* = 25)	Controls (*n* = 17)	A-T ≥12 years (*n* = 15)
GH	1.33 ± 1.87	2.45 ± 4.71	1.04 ± 1.98
IGF-1	198.1 ± 142.1**	327.9 ± 149.3	254.4 ± 138.4
IGF-BP3	4.51 ± 1.35	5.46 ± 1.51	4.94 ± 1.38
Cortisol	13.79 ± 5.17***	21.07 ± 5.05	15.29 ± 5.66*
DHEAS	141.3 ± 127.7***	266.8 ± 87.94	195.5 ± 131.5*
TSH	2.7 ± 1.2	2.51 ± 1.14	2.53 ± 1.22
Vitamin D	18.08 ± 10.09	24 ± 9.55	12.64 ± 6.66**

The data are shown as the means ± SD

Significant differences: *p < 0.05; **p < 0.01; ***p < 0.001

Interestingly, vitamin D levels were significantly lower in the group of A-T patients who were over 12 years of age compared with the controls (A-T (*n* = 14) 12.64 ± 6.66 ng/ml, controls (*n* = 17) 24 ± 9.54 ng/ml; *p* < 0.001). Of the 24 A-T patients, 11 (45.8 %) had vitamin D levels <20 ng/ml, and five had levels <10 ng/ml. All five patients with absolute vitamin D deficiency were over 12 years of age and were wheelchair-bound.

We correlated age, BMI, PhA and muscle strength to patient hormone levels (Table 4). Significant correlations could be established for cortisol, DHEAS and IGF-1.

**Table 4:** Correlations among A-T patients and controls (*n* = 41)

Variable	Mediator	r	*p*-value
PhA	Age	-0.1903	n.s.
	DHEAS	0.4566	<0.01
	Cortisol	0.4314	<0.01
	IGF-1	0.2918	n.s.
	IGF-BP3	0.1649	n.s.
	Vit. D	0.2430	n.s.
FFM	Age	0.8723	<0.0001
	DHEAS	0.7269	<0.0001
	Cortisol	0.6247	<0.0001
	IGF-1	0.4492	<0.01
	IGF-BP3	0.2564	n.s.
	Vit. D	0.06553	n.s.
BMI	Age	0.6597	<0.001
	DHEAS	0.6557	<0.0001
	Cortisol	0.4622	<0.01
	IGF-1	0.4689	<0.01
	IGF-BP3	0.2275	n.s.
	Vit. D	0.01586	n.s
Manual muscle strength	Age	0.6510	0.001
	DHEAS	0.6264	<0.0001
	Cortisol	0.5515	<0.001
	IGF-1	0.4118	<0.01
	IGF-BP3	0.3241	<0.05
	Vit. D	0.2725	n.s.

## Discussion

Poor weight gain, small stature, progressive dystrophy and altered muscle mass are unique features of chromosomal instability syndromes, such as A-T, Fanconi anemia, Nijmegen breakage syndrome, and Werner syndrome. Over time, fatigue and cachexia lead to reduced lung ventilation, decreased quality of life and shortened life expectancy [25]. Because the prevalence of alterations in body composition, muscle strength and hormonal status has not been well described for chromosomal instability syndromes, we performed a detailed investigation of these characteristics in patients with A-T.

The prevalence of cachexia and muscle wasting has been underestimated in relation to aging, chronic disease and cancer, although these conditions lead to high disability and mortality rates [26]. The current study demonstrates that structural differences in body constitution beyond short stature, low weight and reduced BMI exist in A-T patients. The reduced FFM in these patients is indicated by their low PhA, BCM and ECM values. Low PhA values reflect diminished numbers of metabolically active cells with lipid bilayer membranes. PhA provides a rough estimate of the FFM quality and quantity and influences BCM and ECM levels [24]. As expected, low PhA values were correlated with BCM, suggesting declined muscle mass. In contrast to their reduced BCM values, A-T patients did not have altered fat tissue or FM.

The PhA value is a particularly relevant indicator of nutritional status [27]. Low PhA values have been associated with poor survival in patients with human immunodeficiency virus infection, chronic renal failure and hepatocellular carcinoma (HCC) [28–30]. In addition to PhA, the ECM/BCM ratio is an established and sensitive index of malnutrition [31]. Interestingly, 100 % of the adolescent A-T patients in the current study showed increased ECM/BCM values, highlighting their severe malnutrition. Our study confirmed recent reports of profound malnourishment in Brazilian [32] and Australian A-T patients, as significant malnutrition was detected in nine of 13 evaluated patients (69 %), including one severely malnourished adult [10].

In analyzing the impact of behavioral, dietary and physical features on body composition in A-T patients, Ross et al. indicated that a high percentage of their patients consumed too little energy, although the results from the appetite questionnaire that was used in their study were classified as normal in most cases [10]. In a study of Brazilian children with A-T, poor nutritional status was observed, and the children affected by A-T had a lower caloric intake than did those in the healthy control group [32]. Interestingly, malondialdehyde, retinol, zinc and beta-carotene levels were normal in these A-T patients [32], most likely because the patients were young children.

The current study is the first to show that decreases in BCM and FFM accompany the impaired manual muscle strength that is characteristic of A-T. Here, compromised manual muscle strength was demonstrated using a hydraulic hand dynamometer. Two probable causes for the observed myopenia include inactivity and the need to sit in a wheelchair from adolescence onward. Our data suggest that the FFM progressively decreases with age (Table 4, *p* < 0.0001, r = 0.7920); however, the pathophysiology underlying this progression is complex. Myopenia can result from immobility, disease, aging and/or poor nutritional status [33]. The four most common reasons for reduced muscle mass are anorexia, dehydration, cachexia and sarcopenia [34]. All of these factors may play a role in muscle wasting in A-T. Declines in corporal resources corresponding to cachexia are in concordance with our results and have been previously described by several authors [8–11, 32].

When considering A-T as a model of premature aging, another possible explanation could be sarcopenia. Sarcopenia is defined as muscle cell involution in response to aging [35] and is correlated with exhaustion, frailty and diminished strength [35]. One potential mechanism for muscle wasting in the elderly is motor unit loss due to the denervation of aging muscle [34]. This process may be translated into cerebellar neurodegeneration and neuromuscular apraxia in A-T, ultimately resulting in the under- or mis-stimulation of muscles and consequent muscle involution [12, 36, 37]. In addition, alterations in central motor conduction have been reported in older children with A-T [38]. In the current study we could also show the increased neurological impairment in older and wheelchair-bound A-T patients who tend to suffer more from dysphagia, immobility and neuropathy. The neurological dysfunction was emphasized by significantly higher ataxia scores compared to specimen whose gait is still preserved. Interestingly, in an autopsy from 1964, Dunn et al. described that “the skeletal muscle exhibited […] mild atrophy of the fibres” [39].

Alternatively, ATM may play a critical role in muscle energy supply and regeneration. In 2011, Consentio et al. demonstrated that ATM promotes glucose-6-phosphate-dehydrogenase expression and thus regulates the pentose phosphate pathway [40]. In ATM -/- cells, this pathway is dysfunctional such that an inadequate amount of the antioxidant nicotinamide adenine dinucleotide phosphate (NADPH) is produced. Two potential causes of muscle wasting can be inferred from this finding: 1.) the vulnerability of all cells, including muscle cells, to oxidative stress; and 2.) the dysregulation of additional signaling pathways, resulting in reductions in muscle mass.

Elevated reactive oxygen species (ROS) levels and upregulated cytokine production may further decrease muscular strength and increase fatigue in A-T patients [9, 41]. In these patients, multiple genes encoding inflammatory proteins, especially the gene encoding Interleukin-8 (IL-8), are significantly upregulated [42]. In support of this idea, our data indicate that C-reactive protein (CRP) expression is strongly correlated with age. Fatigue and cachexia often occur together and are more pronounced in older A-T patients who are wheelchair- bound. As a result, these patients may be at greater risk for vitamin D deficiency due to inactivity, low sunlight exposure and/or low oral vitamin D intake. Interestingly, osteoporosis has already been described in Atm -/- knockout mice [43].

In the present study, we confirmed that the majority of our A-T patient cohort exhibited altered IGF-1 levels. This finding is not entirely novel; it has recently been described by both our group and others [8, 9, 11, 44–46]. Although IGF-1 levels are reduced in A-T patients, the temporal and/or causal relationship between low IGF-1 levels and muscle wasting and fatigue is unclear. A-T patients present with an imbalance between catabolic and anabolic steroid metabolism, which appears to be related to the loss of muscle strength and the development of cachexia.

A novel association between muscle strength and the adrenal steroids cortisol and DHEAS (both released from the adrenal cortex) was revealed in this study. On the one hand, low DHEAS and cortisol concentrations might result from decreased adrenal steroidogenesis in the zona reticularis [47]. On the other hand, our findings are concordant with a Canadian autopsy report in which atrophic lightweight adrenals were found in a 17-year-old Caucasian female with A-T [39]. Therefore, our data suggest that early adrenal involution/depletion leads to low DHEAS and cortisol levels in A-T patients.

The existence of a connection between cachexia and hormonal dysfunction has been established by many authors in studies of various diseases. Wasting affects the hormonal balance of the adrenocortical system [48]. DHEAS is a central hormone for entry into puberty and physical development. Reduced DHEAS levels may partly explain the delayed pubertal development, poor weight gain and lack of growth spurt in adolescence observed in A-T patients. Furthermore, decreased DHEAS plasma levels have been reported as an adverse prognostic marker in chronic heart failure [49].

In 2012, Menotta et al. reported that dexamethasone induced a truncated ATM protein variant which partly replaces the missing effects of ATM kinase activity in Atm -/- cells [50]. In addition, a proof of concept study confirmed a positive effect of dexamethasone treatment in A-T patients. Monthly infusions of autologous erythrocytes-delivered dexamethasone led to significant improvement of neurological symptoms [51]. The underlying mechanism of the dexamethasone treatment may be difficult to explain. In part it may be related to restored kinase activity. Alternatively, cortisol shortage indicating an early involution of the adrenal glands may be overcome by dexamethasone replacement. Probably, a phase 3 trial will elucidate the positive effects of erythrocytes-delivered dexamethasone in a larger group of A-T patients in the near future [52].

## Conclusion

In conclusion, there is an urgent need for improved and earlier assessments of nutritional problems in A-T patients. BIA is an inexpensive and relatively easily applied tool for the analysis of body composition, as it detects early indicators of qualitative malnutrition. Although single body compartments tend to be under- or overestimated using this method, BIA can still provide an approximate assessment of nutritional status. In the future, poor nutritional status, muscle wasting and fatigue in A-T patients must be treated more aggressively.

## Abbreviations

A-T: ataxia-telangiectasia
ATM: ataxia-telangiectasia mutated
BMI: body mass index
IGF-1: insulin-like growth factor 1
IGF-BP3: insulin-like growth factor-binding protein 3
GH: growth hormone
TSH: thyroid-stimulating hormone
DHEAS: dehydroepiandrosterone sulfate
SD: standard deviation
ROS: reactive oxygen species
IL-8: interleukin-8
BIA: bioelectrical impedance analysis
PhA: phase angle
FFM: fat-free mass
BCM: body cell mass
ECM: extracellular matrix
FM: fat mass
HCC: hepatocellular carcinoma
NADPH: nicotinamide adenine dinucleotide phosphate
CRP: C-reactive protein
LMS: least median of squares
WHO: World Health Organization
AFP: Alpha-fetoprotein
